# Supplementary material for: Psychometric properties of the Persian version of the hospitalized older adults’ dignity scale for measuring dignity during acute hospitalization
Source: PLoS One. 2025 Sep 25;20(9):e0332036. doi: 10.1371/journal.pone.0332036 (PMC12463278; doi:10.1371/journal.pone.0332036)
Supplement: S1 File — (DOCX) [file pone.0332036.s001.docx]

**Persian version of the Hospitalized Older Adults' Dignity Scale (HOADS)**

**عبارات زیر تجربه بیماران از کرامت و/یا مراقبت با کرامت در طول بستری شدن در بیمارستان حاد را توصیف می‌کنند. بر اساس بستری شدن فعلی شما در بیمارستان، لطفاً میزان صحت هر عبارت را برای خود مشخص کنید. برای هر عبارت، گزینه‌ای را انتخاب کنید که بهترین توصیف از تجربه شما باشد:**

**0 = برای من صدق نمی‌کند**

**1 = هرگز**

**2 = گاهی**

**3 = اغلب**

**4 = همیشه**

| گویه ها | 0 | 1 | 2 | 3 | 4 |
| --- | --- | --- | --- | --- | --- |
| 1. ارائه‌دهندگان خدمات بهداشتی من را در بحث‌های مربوط به مراقبتم درگیر می‌کنند. |  |  |  |  |  |
| 2. ارائه‌دهندگان خدمات بهداشتی من را در تصمیم‌گیری‌های مربوط به مراقبتم درگیر میکنند. |  |  |  |  |  |
| 3. ارائه‌دهندگان خدمات بهداشتی به انتخاب‌های من در مورد مراقبتم احترام می‌گذارند. |  |  |  |  |  |
| 4. ارائه‌دهندگان خدمات بهداشتی اطلاعات کافی درباره وضعیت سلامتی‌ام به من می‌دهند. |  |  |  |  |  |
| 5. ارائه‌دهندگان خدمات بهداشتی اطلاعات کافی درباره درمانم به من می‌دهند. |  |  |  |  |  |
| 6. ارائه‌دهندگان خدمات بهداشتی اطلاعات کافی درباره داروهایم به من می‌دهند. |  |  |  |  |  |
| 7. ارائه‌دهندگان خدمات بهداشتی به من توجه می‌کنند وقتی که صحبت می‌کنم. |  |  |  |  |  |
| 8. ارائه‌دهندگان خدمات بهداشتی قبل از انجام هر گونه پروسیجری بر روی من، اجازه من را می‌گیرند. |  |  |  |  |  |
| 9. ارائه‌دهندگان خدمات بهداشتی به انتخاب من برای درگیر کردن خانواده‌ام در مراقبتم احترام می‌گذارند (مثلاً در حمام کردن و استفاده از توالت). |  |  |  |  |  |
| 10. ارائه‌دهندگان خدمات بهداشتی در زمان نیاز به کمک، کمک رضایت‌بخشی ارائه می‌دهند. |  |  |  |  |  |
| 11. ارائه‌دهندگان خدمات بهداشتی هنگام بحث در مورد مسائل مربوط به من، حریم خصوصی را رعایت می‌کنند. |  |  |  |  |  |
| 12. ارائه‌دهندگان خدمات بهداشتی هنگام ارائه مراقبت، حریم خصوصی را رعایت می‌کنند. |  |  |  |  |  |
| 13. ارائه‌دهندگان خدمات بهداشتی با همدلی با من رفتار می‌کنند. |  |  |  |  |  |
| 14. ارائه‌دهندگان خدمات بهداشتی به باورهای مذهبی من احترام می‌گذارند. |  |  |  |  |  |
| 15. ارائه‌دهندگان خدمات بهداشتی به نیازهای من به موقع پاسخ می‌دهند. |  |  |  |  |  |
