# Supplementary material for: Psychometric properties of the Persian version of the hospitalized older adults’ dignity scale for measuring dignity during acute hospitalization
Source: PLoS One. 2025 Sep 25;20(9):e0332036. doi: 10.1371/journal.pone.0332036 (PMC12463278; doi:10.1371/journal.pone.0332036)
Supplement: S2 File — (DOCX) [file pone.0332036.s002.docx]

**Original version of the Hospitalized Older Adults' Dignity Scale (HOADS)**

The following statements describe patients' experience of dignity and/or dignified care during acute hospital admission. Based on your current hospital admission, please indicate the extent to which each statement applies to you. For each statement, please choose the option that best describes your experience: 0 = not applicable to me, 1 = never, 2 = sometimes, 3 = often, 4 = always.

| Items | 0 | 1 | 2 | 3 | 4 |
| --- | --- | --- | --- | --- | --- |
| 1. HCPs include me in discussions about my care. |  |  |  |  |  |
| 2. HCPs involve me in decisions about my care. |  |  |  |  |  |
| 3. HCPs respect my choices about my care. |  |  |  |  |  |
| 4. HCPs provide me with adequate information about my health condition. |  |  |  |  |  |
| 5. HCPs provide me with adequate information about my treatment. |  |  |  |  |  |
| 6. HCPs provide me with adequate information about my medications. |  |  |  |  |  |
| 7. HCPs pay attention to me when I speak. |  |  |  |  |  |
| 8. HCPs seek my permission before performing any procedures on me. |  |  |  |  |  |
| 9. HCPs respect my choice for my family to be involved in my care (e.g. bathing and toileting). |  |  |  |  |  |
| 10. HCPs provide satisfactory assistance when I need help. |  |  |  |  |  |
| 11. HCPs provide privacy when discussing issues about me. |  |  |  |  |  |
| 12. HCPs provide privacy when providing care. |  |  |  |  |  |
| 13. HCPs treat me with compassion. |  |  |  |  |  |
| 14. HCPs show respect for my religious beliefs. |  |  |  |  |  |
| 15. HCPs respond to my needs in a timely way. |  |  |  |  |  |
