# Supplementary material for: Psychometric properties of the Persian version of the hospitalized older adults’ dignity scale for measuring dignity during acute hospitalization
Source: PLoS One. 2025 Sep 25;20(9):e0332036. doi: 10.1371/journal.pone.0332036 (PMC12463278; doi:10.1371/journal.pone.0332036)
Supplement: Attachment — Submitted filename: Response to Reviewers.docx [file pone.0332036.s004.docx]

**PLOS ONE**

**Academic Editor**

**Dear Professor Noushin Kohan,**

       We appreciate your consideration of the revised version of our manuscript, titled "**Psychometric properties of the Persian version of the hospitalized older adults' dignity scale for measuring dignity during acute hospitalization**." We are grateful to the editor and reviewers for their insightful feedback, which has significantly enhanced the quality of our work. Their constructive comments have guided us in refining the manuscript, and we have meticulously addressed each suggestion. A detailed explanation of the changes made in response to the reviewers' concerns is provided point by point in the following pages, with all modifications highlighted in yellow within the manuscript. We hope these revisions meet the requirements for the manuscript's acceptance for publication in *PLOS ONE*.

We are looking forward to hearing from you.

Sincerely,

Reza Nemati-Vakilabad

| **Reviewer 1** | | | |
| --- | --- | --- | --- |
| **No.** | **Reviewer Comments** | **Response** | **Page** |
| 1 | Page 6 Translation procedure, it may be useful to put in the citations for the HOADS by Dr. Abdul-Ganiyu Fuseini, and the citation for the WHO guidelines. | We sincerely appreciate your valuable suggestion regarding the translation procedure section. As recommended, we have now included the appropriate citations for the HOADS by Dr. Abdul-Ganiyu Fuseini, as well as the relevant WHO guidelines.  Again, we sincerely appreciate you for all of your valuable comments to improve the manuscript. | 6 |
| 2 | On page 7, Content validity, were the same panel of experts used in the qualitative assessment used for the quantitative evaluation? | We sincerely appreciate your insightful question regarding the expert panel.  Yes, the same panel of experts who participated in the qualitative face and content validity assessment was also involved in the quantitative evaluation (CVR and CVI). This consistency ensured methodological coherence throughout the validity assessment process. | 10 |
| **Reviewer 2** | | | |
| 1 | The discussion section could provide more comparative insights with similar psychometric validation studies in other languages or settings. | We sincerely appreciate your valuable suggestion regarding comparative analysis with other validation studies. We would like to respectfully note that our study represents the first psychometric evaluation of the HOADS in any language other than the original English version. As such, while we were unable to compare our findings with other cross-cultural validations (as none exist), we have thoroughly compared our results with those of the original instrument's development study.  Again, we sincerely appreciate you for all of your valuable comments to improve the manuscript. | 13-16 |
| 2 | A brief explanation of potential limitations (e.g., cultural biases in self-reported dignity assessments) would enhance the study’s credibility. | We sincerely thank you for your valuable and insightful comments, which have helped improve the quality and clarity of our manuscript. We have carefully addressed all the suggestions, and we believe the revised version has been strengthened as a result.  We greatly appreciate the time and effort dedicated to reviewing our work. | 16-17 |
| 3 | Future research directions could be elaborated further, including potential applications of the HOADS-P in different healthcare settings beyond acute hospitalization. | We sincerely appreciate your insightful suggestion regarding future research directions. In response to this valuable comment, we have expanded the Conclusion section to include additional potential applications of the HOADS-P in various healthcare settings beyond acute hospitalization | 17 |
